# Telephone consulting for ‘Personalised Care and Support Planning’ with people with long-term conditions: a qualitative study of healthcare professionals’ experiences during COVID-19 restrictions and beyond

**DOI:** 10.1186/s12875-024-02443-z

**Published:** 2024-05-31

**Authors:** Sharon McCann, Vikki A. Entwistle, Lindsay Oliver, Nick Lewis-Barned, Rebecca Haines, Alan Cribb

**Affiliations:** 1 Health Services Research Unit, University of Aberdeen, Aberdeen, AB25 2ZD Scotland, UK; 2 School of Divinity, History, Philosophy and Art History, University of Aberdeen, Aberdeen, Scotland, UK; 3 Year of Care Partnerships, Northumbria Healthcare NHS Foundation Trust, Ashington, Northumberland, UK; 4 Glenpark Medical Centre, Gateshead, UK; 5 Centre for Public Policy Research, King’s College London, London, UK

**Keywords:** Healthcare professionals’ experiences, Long-term conditions, Personalised care and support planning, Person-centred care, Primary care, Qualitative interviews, Self-management support, Telephone consulting.

## Abstract

**Background:**

Personalised Care and Support Planning (PCSP) replaces conventional annual reviews for people with long-term conditions. It is designed to help healthcare professionals (HCPs) and patients engage in conversations as equals and collaboratively plan actions oriented to each patient’s priorities, alongside biomedical concerns. Little is known about how the shift to remote consulting initiated with COVID-19 restrictions has impacted PCSP.

**Aim:**

To investigate HCPs’ experiences of conducting PCSP conversations remotely and consider implications for the fulfilment of PCSP ambitions as remote consulting continues beyond COVID-19 restrictions.

**Methods:**

19 semi-structured interviews with HCPs in England and Scotland; interpretive analysis.

**Results:**

HCPs’ accounts made clear that COVID-19 restrictions impacted multiple aspects of PCSP delivery, not just the mode of conversation. Broader disruption to general practice systems for gathering and sharing information ahead of PCSP conversations, and moves to ‘wide window’ appointment times, made it harder for patients to be prepared for PCSP conversations. This constrained scope to achieve PCSP ambitions even with the best professional communication skills. Most remote PCSP conversations were conducted by telephone. In the absence of visual communication with patients, it was sometimes harder to achieve the ambitions of PCSP conversations, including to balance patient and professional agendas, fulfil key planning activities, and foster a relational ethos of equal, collaborative partnership. The challenges were particularly severe when working with new patients and people with complex clinical and social problems. Although options for telephone appointments now offer valued flexibility, sustained experience of struggling to achieve PCSP ambitions via remote consulting led some HCPs to lower their standards for judging a “good” PCSP conversation, and to diminished professional satisfaction.

**Conclusions:**

There are significant challenges to fulfilling the ambitions of PCSP via telephone, especially when preparatory support is limited. This study provides grounds for scepticism about how compatible telephone appointments can be with this person-centred model of working, especially for people who are socially disadvantaged and live with complex health conditions. These threats to the provision of person-centred support for people with long-term conditions warrant careful attention going forward if the PCSP model and its benefits are to be sustained.

**Supplementary Information:**

The online version contains supplementary material available at 10.1186/s12875-024-02443-z.

## Background

With high and rising levels of long-term health conditions in many countries, there has been an emphasis on supporting people to self-manage their health [1–3]. Support for self-management is intended to improve health while reducing reliance on health service provision, by enabling people to manage their health conditions on a daily basis. There is often also an ambition that support should be ‘person-centred’ and oriented to help people not just to survive but to thrive - to live well with their conditions [4].

Person-centredness has been variously characterised, reflecting the plurality of attitudes and behaviours that can be part of ‘treating people as persons’ – at least of not disrespecting or neglecting important aspects of people’s humanity [5, 6]. Supporting people with long-term health conditions often requires, among other things, attention to people’s autonomy and overall wellbeing (recognising that patients do most of the work of managing these conditions, daily and over the long-term). Care is also needed to ensure that healthcare systems’ concerns with biomedical indicators of health do not preclude attention to people’s broader concerns and personal priorities [5, 6].

Care planning was first introduced into the UK with the 2001 National Service Framework for diabetes and was piloted across 3 health communities (Primary Care Trusts) between 2007 and 2011 [7]. Personalised Care and Support Planning (PCSP) was then extended to include other (and multiple) long-term conditions, and introduced into UK primary care organisations from 2011, as a person-centred approach to support self-management of long-term conditions [8].

Personalised care has been included within UK health policy for more than 20 years and is considered key to enhancing the quality and safety of care with the involvement and engagement of people via shared decision making around health care choices and the promotion of self-management. Year of Care Partnerships® [YOCP] is an NHS organisation dedicated to the implementation of personalised approaches to care and has worked with over 40 organisations across England and Scotland including commissioning groups, individual and groups of practices. YOCP supports a network of quality assured trainers and a community of practice including trainers, clinical champions and exemplars to share and develop learning related to PCSP and person-centred care more generally.

**Fig. 1:**
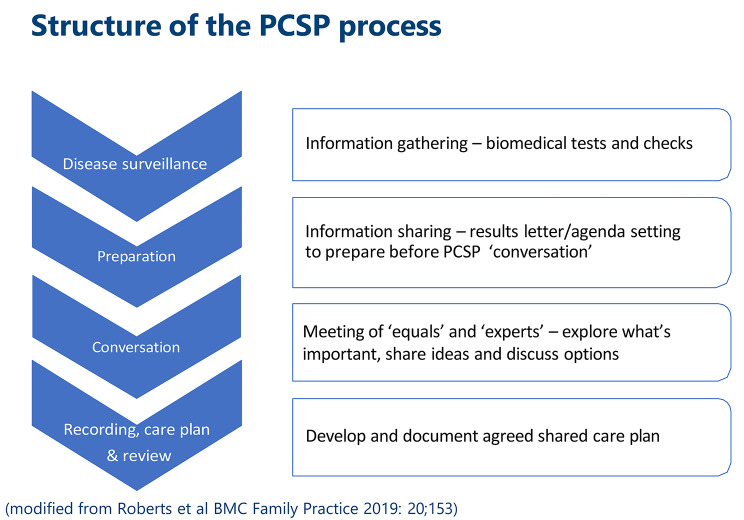
Structure of the PCSP approach

PCSP provides a systematic approach to foster better conversations between patients and PCSP-trained HCPs. It is carefully designed to make it easier for HCPs and patients to work collaboratively, bringing together traditional clinical issues and the person’s lived experience in a solution focused, forward-looking conversation. While emphasising both biomedical and personal aspects of care, PCSP is underpinned by a focus on ‘people not diseases’ [8]. The PCSP approach recognises the person as the main agent living with and managing their conditions [8]. Underpinned by key relational aspects of respect and care, the intentions behind PCSP are to prioritise the values and capabilities that matter to individuals. To support planning around these priorities, PCSP conversations are typically conducted annually, within an extended appointment ‘slot’, with the overall purpose of developing or refining plans that support people to live well on their own terms [8]. To prepare patients for a PCSP conversation, they are sent a letter in advance that includes an agenda setting prompt, which summarises any routine biomedical indicators of their health status and encourages them to think and note down what they would like to discuss during the conversation. This key preparatory stage is intended to help make it possible for patients and HCPs to meet as equals. Figure 1 illustrates an overview of PCSP structure.

With the introduction of COVID-19 regulations in the UK in March 2020, the UK government instructed general practices to conduct all consultations remotely unless there was an urgent need for an in-person appointment [9]. This resulted in the rapid and widespread use of remote consulting [10–12]. PCSP conversations, like most primary care appointments, could, for a while, only be conducted remotely (by telephone or internet video communication). Research indicates limited use of video and greater reliance on telephone for remote consultations during the pandemic. As restrictions eased, the number of general practice appointments conducted by video remained low [12]. NHS data from July 2022 show nearly 30% of primary care appointments were delivered via telephony, with 65% face-to-face [13]. Most recent figures from NHS England show that in November 2023, 67.9% of general practice appointments occurred face-to-face [14]. With the ongoing use of remote care approaches in primary care, concerns have been raised about adverse impacts on continuity of care, particularly for patients with long-term or complex conditions [15].

The extent to which telephone consulting has continued since restrictions on in-person consultations were lifted is part of a changing landscape for healthcare consulting generally. Recent NHS plans indicate an intention to keep remote consulting as an important modality for UK general practice [16, 17]. Therefore, it is important to understand any challenges to conducting PCSP remotely, and when and how it can work well, if the PCSP model and ethos are to be sustained.

Our aim in this study was to investigate HCPs’ experiences of conducting PCSP conversations remotely following the introduction of COVID-19 restrictions and to consider the implications of their perspectives for the fulfilment of PCSP ambitions as the use of remote consulting continues beyond COVID-19 restrictions.

## Methods

This was a qualitative interpretive research study focused on understanding the experiences and views of HCPs conducting remote PCSP during and after the restrictions on face-to-face consulting imposed due to COVID-19. We sought to interview a diverse sample of HCPs from general practices in England and Scotland who had been using PCSP as characterised and taught by YOCP before the pandemic, and who were involved in a shift to conducting PCSP conversations by telephone and/or video.

### Sampling and recruitment

We sought to include HCPs working in a mix of roles (especially GPs, nurse practitioners and practice nurses) and set a sample size target of approximately 16–25 interviews, guided by the pragmatic principles of information power [18].

In line with ethics approval, local PCSP leads were identified by the YOCP National Director via:- (a) YOCP mailing list of PCSP site leads for geographical areas; and (b) YOCP mailing list of practices and individuals who were trained within the YOCP programme. The Director sent an email invitation and a participant information leaflet [Additional File 1] to local PCSP leads asking: (a) whether they would participate themselves; and (b) to share email invitations (to opt-in) with eligible colleagues. To avoid undue pressure to participate, the invitations from the YOCP National Director requested anyone who was potentially interested to ‘opt-in’ by directly contacting a university-based researcher (SM).

The researcher (SM) replied to those who made contact to discuss the study and, if appropriate, arranged an interview. HCPs were informed that the study aimed to learn from their experiences of in-person and telephone and/or video PCSP conversations, including any challenges and adjustments made in the shift from in-person consulting.

Recruitment to the study was very challenging in the context of COVID-19 and ongoing pressures on health services, even as restrictions on meeting in person eased. Email responses from some HCPs who declined participation highlighted severe staff shortages and burn out, with no capacity to participate in non-essential research activities. Added to this, some HCPs who initially confirmed willingness to participate in the study were then persistently ‘out of office’ on sick leave, and several needed to reschedule interviews due to work demands. Two HCPs ‘opted in’ after other participants passed on study details to them after their own interviews.

Our final sample comprised nineteen HCPs from 14 general practices (England n = 10; Scotland n = 4). They included: 9 practice nurses (including 1 YOCP trainer); 5 nurse practitioners (including 3 YOCP trainers and one advanced nurse practitioner); 1 pharmacist; 3 GPs (including 1 YOCP trainer and co- author), and 1 YOCP National Lead (and co-author). YOCP trainers had completed additional ‘train the trainer’ courses and had experience of introducing new colleagues and supporting implementation of PCSP.

Two co-authors (LO and RH) were also interviewees. After careful consideration of using their interviews only for pilot purposes, the team agreed that the views and experiences expressed in those interviews were not outliers and warranted inclusion in the interpretive analysis. However, we have quoted them sparingly and added ‘co-author’ to the relevant quotation attribution. Informed consent was digitally recorded before interviews commenced.

### Interviews

Interviews were conducted between August 2021-March 2022 by SM. They were conversational in style, and supported by a topic guide [Additional File 2]. Broadly, the researcher prompted discussion regarding HCPs’ understanding and enactment of PCSP, what worked well/less well by telephone/video, and considerations for continued use of telephone/video PCSP consulting. Interviews ranged from 34 min to 65 min, with a mean interview time of 52 min. Interviews were recorded using Microsoft Teams.

### Data management and analysis

Interview recordings were transcribed verbatim, and transcripts checked and corrected against audio recordings by SM. Data management and preliminary analysis was facilitated by NVivo (v12) text management software, although as explained below, we developed our final analysis using summary charts created in Microsoft Word.

We used an interpretive approach to analysis supported by team discussions to help develop and check the sense we were making from the interview data. As a team, we brought considerations from a variety of clinical (NLB, RH, LO), academic (AC, SM, VE) and patient/carer (all) experiences. We are all interested in PCSP as an approach to enable HCPs to use their biomedical and broader professional expertise to support people with long-term conditions to live (and die) well on their own terms.

Our analytic methods belong to the thematic analysis family [19]. SM initially read through the first available transcripts and in discussion with VE, developed an initial coding framework that was organised into 6 broad categories covering: (a) the different stages of care and support planning; (b) changes associated with the shift to remote consulting; (c) the various characteristics of healthcare professionals and patients that seemed to matter in the shift; (d) evaluative judgements of PCSP consultations (including statements about the purpose(s) of PCSP and how well they were fulfilled); (e) adaptations to PSCP conversations that were made or proposed for remote as contrasted with in-person consultations; and (f) other text that seemed important but did not readily fit within the above 5 categories.

After using this initial framework to code several interview transcripts, and following research team discussions around the transcripts, the framework and our developing understanding, we decided that the coding framework was not fully capturing insights to some of our key research questions when reading and comparing the transcripts. We therefore pivoted our approach and, while continuing to read and reflect on each transcript in its entirety, we progressed the development of our interpretive themes by preparing summary analytic charts in which each participant was allocated a row and relevant points and quotation fragments were recorded (by SM) under four column headings. The four headings related to: (A) the HCP’s account of the purpose(s) of PCSP and differences between PCSP and non-PCSP consultations; (B) features of working context as affecting PCSP (including changes to organisational processes with the introduction and easing of COVID-19 restrictions over time); (C) differences (including adjustments made by HCPs) when PCSP conversations are conducted by phone as contrasted with face to face; and (D) evaluative comments relating to remote and face-to-face PCSP.

The four column chart entries for each participant often extended over several pages (in landscape orientation – see example page in Additional File 3) but this organised summarising still allowed us to: (a) read across rows to appreciate (for example) the trajectories of individual HCP’s experiences and HCP’s apparent fidelity to PCSP principles and approaches, and to consider how their evaluative judgements about PCSP by telephone related to their reported experiences, the adjustments they made, and the purposes of CSP that they explicitly emphasised and implicitly neglected in the enactments they described; and (b) read down columns to consider variation among HCPs and develop and check a sense of the balance of experiences and evaluations within the sample. Columns B, C and D particularly underpinned the development of the three main interpretive themes reported below. Feedback from peer reviewers has also supported the refinement of our presentation.

The quotations we use to illustrate our themes are presented verbatim, but we have deleted hesitation sounds and spoken word repetitions. Where we have omitted words to improve clarity, this is indicated by ‘…’.

## Results

All participants reported an initial suspension of PCSP activities when COVID-19 restrictions were introduced in March 2020, and a (usually ‘slow’) bringing back of ‘some form’ of PCSP (mostly via remote consulting) subsequently. All reported using almost exclusively telephone, rather than video, for remote PCSP conversations. Most attributed this to patient preference and/or concern about digital exclusion, although some acknowledged their own reticence to use video due to concerns about technical skills for that and relative familiarity with telephone. At the time of their interviews (August 2021 – March 2022) all participants were still holding some if not most PCSP conversations by telephone.

We report our analysis within three main interpretive themes, and 10 sub-themes (see Table 1). The overarching narrative, with the three main themes noted, is as follows. The shift to remote consulting at the start of the COVID-19 pandemic impacted several aspects of PCSP. Broader disruption to healthcare practices affected the systems underpinning core PCSP processes and led to reduced staff and patient preparedness for PCSP conversations (theme 1). This constrained the extent to which the ambitions of PCSP could be achieved even with the best PCSP communication skills. The loss of visual communication with patients significantly undermined the fulfilment of ambitions within PCSP conversations by telephone (theme 2). All HCPs illustrated ways in which the experience and significance of changes, and associated losses and challenges could be much more problematic for some patients than others. There was also, not surprisingly, some variability in the ways HCPs reported responding to the challenges and in the emphases within their evaluative assessments of PCSP by telephone. While all saw some value in telephone consulting for the future, their assessments indicate reasons to be cautious about using this routinely for PCSP conversations (theme 3).

**Table 1:** Summary of analytical themes and sub themes

Theme	Subthemes
1. Reduced preparedness for PCSP conversations	1.1 Disruptions to preparatory information gathering and information sharing 1.2 ‘Wide window’ appointment slots and patients ‘not in the right headspace’ 1.3 Reduced third party support for patient participation in PCSP
2. Ambitions undermined within PCSP conversations	2.1 Getting off to a slower start and working with a more limited picture 2.2 Losing balance between patient’s and professional’s agendas 2.3 Missing out on goal-setting and action-planning 2.4 Finding relationship building harder
3. Taking stock	3.1 Evaluative assessments of telephone-based PCSP 3.2 Valuing options for modes of conversation 3.3 Concerns about standards: striving to preserve what matters

### Theme 1: Reduced preparedness for PCSP conversations

#### Disruptions to preparatory information gathering and information sharing

All study participants highlighted that support for patients to be prepared for their conversation is one of PCSP’s key characteristics. The PCSP practice of sharing of biomedical test results and agenda setting prompts in advance was described in significant, positive contrast with previous annual disease review arrangements. For example:

*“[Previously] it was like, going to the headmaster for them to read your report to you. But the thing about the [P]CSP was, that the whole implementation of somebody getting some information, getting some results, getting something to prompt thinking beforehand, so that they felt they at least they had some leverage, seemed to me the thing that we’d been looking for” [NP 03]*.

Although arrangements within their practices varied, most HCPs reported that COVID-19 restrictions significantly disrupted processes established to support the Disease Surveillance (information gathering) stage of PCSP, limiting scope for both patients and HCPs to enter a PCSP conversation prepared with relevant, up-to-date information about the patient’s condition. Patients further missed out when the stalling of systems for the Preparation (information sharing) stage meant they were not sent summaries and prompts to reflect on whatever information had been gathered.

Information gathering, including weight measurement, breathing tests, blood pressure and blood tests, had previously all been done in-person at practices. After initial suspensions of PCSP in March 2020, HCPs described information gathering being re-commenced at varying times and to different extents, sometimes with patients self-reporting data where possible, mostly via SMS (short message service) platforms. HCPs also noted significant limitations and variations in patients’ abilities to measure biomedical markers and communicate these or symptom reports, not least because many could not afford, or lacked skills and confidence to use, healthcare and communication technologies such as blood pressure monitors, internet questionnaires and smartphones:

*“…it seems extraordinary in this day and age, but a lot of our patients, they don’t have …mobile phones, … where we are here it’s quite a deprived area and I was shocked at how many young people didn’t have that kind of technology available to them….”[ NP 05]*.

Reduced information gathering and reliance on patients’ self-reports continued in most participants’ practices as COVID-19 restrictions eased, and in some cases to the end of our data collection period (March 2022). HCPs attributed this in part to some patients preferring to avoid in-person encounters, including due to continued risk from COVID-19. Some also said that their practice teams continued to lack administrative capacity to initiate and synchronise information gathering. Some HCPs reported an ongoing lack, or shortfalls in timeliness of the sharing of results and agenda-setting prompts in advance of PCSP conversations (attributed again to limited administrative capacity and sometimes a low priority being given to PCSP by practice leaders).

Whatever the underlying reasons, the PCSP principle that patients and HCPs should have the same preparatory information to support their meeting ‘as equals’ in the PCSP conversation, was undermined.

#### ‘Wide window’ appointment slots and patients ‘not in the right headspace’

Scope for patients to join PCSP conversations in a state of preparedness was also lost when practices modified appointment scheduling as they shifted to remote consulting in the pandemic context. HCPs described how, when PCSP conversations were reintroduced by telephone, patients were given ‘wide time windows’ within which they could expect a call from a healthcare professional (e.g. 8am-12 noon or even 8am to 6pm), rather than specific appointment times. Some HCPs said they appreciated how ‘wide window’ slots could in facilitate ‘flex’ in the system and enable them to deal with emergencies and prioritise other tasks as they saw fit. But they also told us about frequent difficulties ‘getting hold of patients’ on the telephone, and about patients often not being ‘in the right head space’ for a PCSP conversation, because when the HCP called, they were busy doing something else:

*“...it depended on where you caught the patient because unfortunately we weren’t giving them specific times when we would ring them, so the patient wasn’t always prepared, so you might catch them when they would be in the shop or would be in a car, or whatever, so it wasn’t always a good, it wasn’t planned so the patient wasn’t prepared for your phone call; they weren’t sitting down in that headspace thinking, “Right, so I’m going to have the consultation with a nurse. This is what I need to bring up, this is what I want to talk to her about.”* [NP 09].

In such circumstances, and being aware that some people lacked a private, safe space from which to talk on the telephone about their health, HCPs described having to ‘work harder’ and decide whether and how to continue the conversation. Sometimes a combination of concerns about the quality of the conversation and the patient’s health condition and needs led them to book another appointment:

*“ so I had a phone call like that yesterday, and the lady just really wasn’t happy speaking because she, was in a shop and she was with friends and it was quite a personal matter and we just left it, we didn’t push it…I just said, “Listen, I’ll book you in for another appointment.”* [PN 14].

In some interviews, we detected not only a sense of professional frustration but also perhaps a tendency to judge patients negatively, with little apparent recognition of the challenges associated with the practice’s expectation that patients wait ready for a telephone call somewhere within a period of several hours.

#### Reduced third-party support for patient participation in PCSP

Some HCPs also noted that it could be harder to co-ordinate the availability of family members, support workers or interpreters for telephone appointments. This too could mean either more time-consuming re-scheduling of appointments or ‘double-handling’ of patients, or less satisfactory PCSP conversations for those who usually benefited from the support of others.

“*often it would take a few phone calls, so, you know, in some ways, you might think it would be easier and quicker on the phone, but often it’s not, because it takes two or three attempts … they would get a son or a daughter involved, so then we’d rearrange it at a time when they could be there and use their phone… to be able to communicate. Em, so yeah, it was difficult and time consuming.” [ NP 05]*.

*“when you’re … dealing with an interpreter, it’s harder to get your feelings across to the patient and it’s harder to understand them as well. So…when you see that patient face-to-face, I might get more from it, but if you put them on a phone call and you put an interpreter on and quite often we have a helpful husband or wife in the background so it’s not a two-way conversation, it’s three-way conversation.” [NP 06]*.

### Theme 2: Ambitions undermined within PCSP conversations

At a basic level, the most obvious differences HCPs noted between telephone-based and face-to-face PCSP conversations were the (inherent) lack of visual communication with patients and the (contingent) shorter time allowances for the telephone-based conversations. (HCPs reported that time allocations for PCSP conversations had been reduced from 20 or 30 min to 15 min or less, for combinations of reasons including staffing pressure, logistical challenges and patients being less willing to speak on the phone). Both these features, but particularly the lack of visual communication, challenged HCPs’ enactments of PCSP conversations. We consider the challenges within 4 sub-themes, three relating to aspects of the PCSP conversational structure, and a fourth focused on PCSP relational ethos.

Structurally, after preliminary greetings, PCSP conversations are supposed to start with attention to the patient’s reflections, concerns and questions. The healthcare professional then introduces any clinical concerns they consider additionally important, and the conversation continues with collaborative discussion about priorities, goal setting, and the development of action plans. The PCSP ethos is supposed to be one of relational equality and collaborative partnership, with HCPs offering non-judgemental support oriented to enabling people to improve or maintain aspects of their lives and wellbeing that matter to them.

For context, it is important to note that the understandings of PCSP evident in participants’ accounts were broadly consistent with YOCP publications and training, although a few participants’ summaries of PCSP and some descriptions of particular consultations suggested more emphasis on standard patient education and biomedical indicators as targets and less on responsiveness to each patient’s needs, perspectives and priorities for wellbeing than might have been expected.

HCPs identified various ways in which telephone consulting, particularly in the context of reduced preparedness for the PCSP conversation could affect their following of the intended PCSP structure, impede their finding a good balance between patients’ and their own priorities, and make it harder to establish the kind of relational ethos aspired to.

#### Getting off to a slower start and working with a more limited picture

Some HCPs noted that without visual aspects of communication, it was harder to orient patients who were not already familiar with PCSP into this different kind of conversation:

*“…for patients that were new to care and support planning, it was harder to, kind of, guide them through that process on the phone without the sort of visual cues and communication skills there.” [NP 05]*.

Some also reflected that their initial questions were more constrained and less effective in opening up a conversation by telephone:

*“with the opening the consultation… if they’re here [in person], I normally would just like say, “Oh, how you doing? How’s things?” or, you know, make some comment and that normally opens up the conversation. But I think not having that face-to-face interaction, you’ve lost initial opening up of, “Oh, you’re looking well,” …you just miss that I found.” [PN 11]*.

When biomedical data relating to long-term conditions was available but had not been pre-shared, conversational time was needed to share this, and the sharing could take longer and be less effective without options for visual support:

*“in a phone consult where ….they’re not aware of their results, I will now spend five minutes going through them, and that can take a bit longer on the phone because you might need to repeat things, or talk it through, rather than just show them a graph and say, “Look, it’s gone up and down and look here it is now, and this is where we want it to be” etc. So, you, you lose some of that, that sort of power of visuals.” [GP 18]*.

Similarly, if a patient in a telephone consultation had forgotten or lost a results and agenda setting prompt letter that had been sent, HCPs could not simply print and hand over a replacement copy or turn their computer screen so the person could view the information there.

For PCSP conversations, as for other kinds of consultation, HCPs particularly missed being able to ‘eyeball’ patients to pick up on often subtle indicators of how they were doing, including (for patients they had seen before) signs of change:

*“I definitely feel physical cues are missed and lost when you are not seeing the patient face-to-face which is vitally important when assessing patients. I feel I have to ask a lot more questions over the phone to ‘paint the picture’ whereas if you had the patient in front of you, you can see how well they are walking or if they use walking aids, you can often assess how frail a person is by looking at them.” [PN 11]*.

The boundary between introductory greetings or conversational openers and the ‘patient’s story’ part of the consultation, in which the patient’s perspective and agenda are more fully heard and explored is often blurred. We turn now to the patient’s story and considerations of how the patient’s and HCP’s priorities are combined.

#### Losing balance between patient’s and professional’s agendas

HCPs expressed various concerns, at least sometimes connected to the loss of preparatory prompting of patients to consider their priorities for discussion, but also in association with the use of telephone and the broader pandemic context that led to that, about patients needing to be reminded to think and tell the HCP about their perspectives on how things were going with their condition(s). Several HCPs suggested that patients tended not to be as open about their story on the telephone as they were in person – perhaps in part because they were not in a space designated for healthcare with relatively little to distract from a consultation focused on them, and perhaps because they thought they had less time to give a response. On the telephone, too, there was no opportunity for HCPs to see and perhaps gently pick up on an issue that a patient had not mentioned when speaking:

*“I mean mental health is a massive thing, and a lot of patients want to talk about mental health, probably more so face to face than on the phone. I feel, like patients maybe wouldn’t feel like, they don’t know how to bring that up, because sometimes I will initiate that conversation if they’re looking a certain way or … they’ll circle the word “mental health” on their paper and I can see that so then I can bring it up, whereas I can’t see what they’ve wrote on their piece of paper when they’re on the phone.” [PN 11]*.

Also, although in some contrast, HCPs experienced challenges with patients sometimes reeling off long lists of concerns – more than could be addressed within one consultation. Some HCPs found it harder to identify patient’s own priorities over the telephone:

*“I think for the conversations in care and support planning, where you are trying to work out what’s important to the patient, I think a face-to-face conversation does enable that much better than certainly a phone consultation.” [GP 13]*.

Some HCPs reported seeing more patients with long lists of concerns when they resumed in-person PCSP conversations. Their explanations (beyond the point that people had struggled generally to access healthcare) also raised concerns about telephone consulting: that patients had sometimes thought an issue not urgent or important enough to raise on the telephone, had worried about lack of privacy or been too embarrassed to raise sensitive issues on the telephone, or had thought the health professional on the telephone was too pressed for time. We heard several examples of face-to-face conversations in which the HCP had been able to pick up subtle cues from body language and explore and ‘get to the bottom of the problem’ in a way that they were sure they would not have been able to do on a phone call.

HCPs recounted various ways of responding to patients who presented long lists of concerns, but most can be summarised as trying to identify and prioritise what was most concerning the patient, and in some cases, holding back their professional concerns for a subsequent appointment. For example:

*“[patients have] a list of things to talk to you about, and a lot of my colleagues have seen that, they literally have ten things they want to talk to you about, which is fine, but, it’s quite challenging, …[I]. feel as if I have to just park, you know, anything that I want to discuss and just hand it over to them….I would…just rebook them for another appointment, because sometimes there’s just too much to do, and not enough time” [PN 19]*.

While these responses to patients’ lengthy lists of concerns can tip the balance of conversation in a way that perhaps neglects the professional’s (typically biomedically informed) concerns about long-term conditions, some HCPs suggested that telephone-based conversations could also tend to lead to the contrasting problem of a health professional’s voice becoming too dominant:

*“I think it’s easy to rabbit on as a healthcare professional on the phone, because you’re phoning, it’s your instinct to, to take control, on the phone…. there is a tendency to revert to type, you know, I think it is very easy to slip into old ways of consulting…because you are staring at your screen as well, whereas in their actual consultation, face-to-face you don’t look at your screen nearly as much and you’ll dip into it if they’re asking you a question but, whereas when you’re on the phone, you’re looking at your screen the whole time, so it’s easy to get distracted.”[PN 08]*.

In part the risk referred to here is that while on the telephone, HCPs spend more time looking at the computer than when they are in a room with a person, and the information they see on that is likely to prompt focus on a biomedical agenda, which in PCSP should be only part of the agenda and considered in the light of what matters to the person. The distraction may also have implications for the relational considerations that we discuss in the sub-theme  'Finding relationship building harder’.

Both of these contrasting problems (needing more time to draw out how a person was doing and needing more time to hear and try to prioritise among a list of concerns) could lead to pressures on time for other key elements of the PCSP conversation, especially when consultation slots had been shortened.

#### Missing out on goal setting and action planning

Most participants said they found it more challenging to engage in collaborative goal setting and action planning within a telephone- compared to an in-person - PCSP conversation. They gave various combinations of reasons, including: time pressures (especially in shortened conversation slots); not being so habituated to, or reminded of, the structure of PCSP conversations in telephone mode; the different ‘dynamics’ of telephone consultation not being so conducive for goal setting and action planning; and not having such an obvious sense of being able to write something together, or of the patient perhaps taking a physical plan away from the conversation:

*“One of the things we definitely don’t do well enough is agreeing an action plan and documenting it. I know this is pretty basic part of [P]CSP, but we still seem to struggle with it… I think doing reviews by phone has made things worse from this point of view, as before COVID the patients would at least have had a handwritten plan to take away … it’s too easy to forget about the outcome when doing conversations by telephone.” [GP 13]*.

Some HCPs seemed keen to supplement telephone PCSP conversations by sending patients additional action-oriented information – for example using SMS messaging and signposting patients to recommended websites:

*“I can text them it as I’m speaking. I’ll be like, “Oh, I’m sending you the information for a diabetic diet. I’m sending you links to…NHS weight loss plans”…and things like that…trying to explain to someone how to use a new type of inhaler over the phone is very difficult, but there’s so many resources out there, like video links, that patients can watch over and over again.” [PN 11]*.

It was difficult to tell from interviews to what extent such patient education efforts were responsive to particular patients’ needs and concerns as ascertained via the PCSP conversation or to what extent they were indicative of HCPs prioritising a relatively standardised professional agenda.

#### Finding relationship-building harder

Most HCPs either commented explicitly or indicated indirectly that relational considerations are very important for the PCSP approach. Some noted particularly that not being able to see or be seen by the patient they were communicating with could make it harder to build rapport and trust by telephone:

“*I don’t know whether you can use the phone to create that trust that face-to-face created… because people remember or recognise you by, by seeing you, by sitting in your room, talking to you… and looking in your eyes as you explain something and, and resonating with that or not, and you just don’t get that by phone*” [GP 18].

There were similar concerns about scope to develop and communicate empathy and reassure patients that they would not be inappropriately judged:

*“I think a lot my communication with people is the non-verbal, I’m trying to convey that I’m not being judgemental and I’m not trying to tell them what to do… But I find that hard to do on the phone.”* [NP 05].

Lacking the usual clues gleaned from patients’ facial expressions and body language, HCPs also felt less able to detect and respond appropriately to patients’ attitudes and emotions by telephone, especially when they were not familiar with the patient. For example, it could be harder to tell whether an expressed agreement was sincere or superficial, to gauge how engaged someone was by a particular topic or suggestion, and to interpret what might be going on in a silence:

*“I think face-to-face, if somebody goes quite silent, it feels quite comfortable to sit and just wait. But on the phone I’m like, “Hello? Hello? Are you still there?” You know, it’s harder just to sit and have a silence on the phone.” [GP 2-co-author]*.

Difficulties achieving the kind of relational ethos aspired to within PCSP were seen to have important implications for practical aspects of care and potentially patients’ health outcomes, as well as being significant in their own right. They can perhaps help explain why HCPs reported being less likely to complete the collaborative processes of goal setting and action planning when PCSP conversations were conducted by telephone:

*“I hate doing over the phone consultations because - it’s about the empathetic relationship between nurse and patient, it’s about shared decision making, it’s about sitting down together as a team, and making a plan. And it’s really hard to do that over the phone because you can’t see the patient.” [PN 04]*.

This same HCP was one of several who expressed concerns to the effect that without visual cues, their PCSP conversations could potentially become more transactional – losing the relational ethos of equality and respectful collaboration at the heart of the approach:

*“it’s a whole different ball game. It changes everybody’s state of play. I think the patients can’t see me, so they, almost it doesn’t feel like it’s a partnership. I feel like, it’s almost feels like a little bit of a ticky box.” [PN 04]*.

Added to this, some HCPs described how they could ‘lose steam’ more quickly when unable to see the patient. Conducting and staying focused within PCSP telephone conversations could be more tiring than conducting them in person:

*“I think being on the ‘phone for a while can be quite draining…Sometimes if I’m on for too long I can get quite easily distracted by other things.” [PN 14]*.

Such deterioration of attentiveness could have negative consequences for the sense of relationship, as well as the balance of conversational content that we discuss in the sub-theme 'Losing the balance between patient’s and professional’s agendas’. 

### Theme 3: Taking stock

#### Evaluative assessments of telephone-based PCSP

When asked for their overall evaluations of telephone PCSP conversations, HCPs gave mixed responses with a variety of reasons that signalled the importance of attending to context when interpreting any particular expression of judgement.

After the loss of PCSP provision at the start of COVID-19 restrictions, PCSP conversations as reintroduced by telephone were seen as ‘better than nothing’, even though the challenges noted above meant some ambitions for PCSP went unfulfilled. Yet the limited extent to which PCSP was re-established in the wake of COVID-19 restrictions and the continued predominance of telephone for PCSP conversations (both variable across practices) gave HCPs causes for concern.

HCPs recognised that conducting a PCSP conversation by telephone could be easier in some situations than others, and could work well for some patients - typically people who were managing well, had no new or complex clinical problems, could communicate easily in spoken English, and were known to the health professional:

*“it’s [telephone PCSP] easier with … people who have already got a plan, they see this as convenient, they see it as a way of accessing some support, they’ve got good verbal skills themselves, and so don’t find any kind of communication difficult, or people I already have that relationship with, those are for me the people who are sort of relatively easy.” [YOCP Lead 01 – co-author]*.

For work with other patients, however, the various challenges threatened a less effective encounter. People who were struggling with multiple health conditions, including frailty, and with social concerns, including poverty, poor housing and recent immigrant status, were considered to be less well served.

The shift to telephone consulting also had downsides for HCPs’ own experiences. Although a few commented positively on the convenience of remote working, the stronger impression from the interviews was that finding lesser scope to fulfil the potential of PCSP by telephone had negative implications for professional fulfilment. This quotation illustrates how a cluster of considerations about process, relationship, effectiveness for patients and professional sense of satisfaction could feature in HCPs’ assessments of a ‘good’ PCSP:

*“it’s based on how they verbalise contentment, they’ve maybe come up with a great plan that’s realistic…. and the conversation flows like a lovely dance, and you….you just feel you’ve made a difference and you’ve done good. It’s like almost like a glow you get when it goes … really well, it’s quite inspirational, really.” [NP 12]*.

We heard several suggestions that not only were these indicators of success less likely to be achieved with telephone-based PCSP conversations, but that HCPs’ expectations of success had been lowered with sustained recent experiences of lesser achievement of PCSP purposes and ambitions. For example:

*“I think previously how I judged a good one would be, if the patient was engaged, I felt we like we had…a conversation that was …flowing both ways, so the patient’s bringing stuff to the table, and I was able to support and listen, that we could work towards some goals …certainly, a good goal-setting session where the patient really was onboard with what you could offer them from your point, and what they could bring to the table, that was how I would measure a good one. Now how I measure a good care planning and support, I think if I can just get the patient to tell me two or three things that they might not have said had I not had those care and support questions to ask them.” [NP 09]*.

HCPs variously recognised that the challenges they experienced and reported as associated with telephone PCSP were not entirely due to the mode of conversation. Rather, they were intertwined with other features of the healthcare system and social circumstances of the time, including shortcomings in processes for preparing patients for conversations, the proliferation of health and wellbeing concerns arising in the pandemic, and public anxieties about the capacity and state of the health service (especially as influenced by negative media reporting). Some HCPs (especially nurses) also acknowledged that their own skills and confidence with telephone consulting had been low when they were first thrown into it, and some also noted that some of the challenges they associated with patients’ circumstances and limited ability or willingness to engage in PCSP activities would still be experienced at least to some extent in face-to-face conversations.

Nonetheless, the difficulties experienced with telephone as a modality suggest a particular need to attend to that.

#### Valuing options for modes of conversation

Having become somewhat accustomed to telephone consulting, including for PCSP, at the time of their interviews, study participants all thought it had a place, and some were careful to avoid saying it was universally inferior:

*“I think that each modality has its following, and place at any one time. I don’t think that face-to-face is always the answer.” [Pharmacist 16]*.

Most HCPs thought that the use of telephone appointments, including for some PCSP conversations, was useful for situations of high demand. Remote consulting also offered flexibility that could help HCPs manage clinical workloads, perhaps especially when patients were given ‘wide window’ time periods within which an HCP would call, and when telephone appointments were generally shorter.

Telephone appointments could also better suit some patients’ preferences, and all HCPs expressed some support for continuing to offer patients an option for telephone PCSP conversations when in-person appointments were also possible. In addition to working well for some people whose health conditions were stable and who were looking for more convenience within a relatively affluent lifestyle (for example, to speak on the phone from their private office rather than taking time off work for an in-person appointment), HCPs also noted that some people who had previously repeatedly defaulted in person appointments perhaps benefited from more contact when it was offered by telephone.

However, there remained a strong sense of the importance of offering in-person PCSP appointments as far as possible. In part this was to help secure the potential benefits of the approach, especially for patients with more complex health and social problems, or new to PCSP. But some HCPs also stressed that patients had been asking for their review appointments to be held in person - and sometimes turning up in person at practice premises even when they had been told it would by telephone:

“*I think on the whole patients have made it very clear that they want to be seen and they don’t want remote consultations.*” [NP 05].

HCPs indicated that this may have been particularly the case for people who took their health concerns and PCSP conversations seriously and, like the HCPs, realised that important affordances for effective support had been lost with the switch to telephone.

#### Concerns about standards: striving to preserve what matters

Depending in part on what was happening in their own practices, HCPs expressed worries about unprepared patients being or becoming ‘the new normal’, about proactively allocated PCSP appointments being used for professionally driven ‘safety netting’ contacts or medication reviews, about consultations designated as PCSP conversations defaulting to more transactional and biomedically dominated encounters, and about losing the value of collaborative, solution-focused planning to address patients’ particular concerns. These kinds of worries led some HCPs to suggest a need for action to preserve the key features and value of PCSP as a person-centred approach:

*“I think….if we don’t take a hold and try and improve, try and focus on making sure that we maintain the centrality of [P]CSP, I think there is a risk, that it will become a brisk medication review on the telephone, and focus more and more on the clinical aspects, on the strictly measurable clinical aspects.” [NP 03]*.

This need went beyond requests for specific training on conducting PCSP conversations by telephone to practice and policy level systems considerations.

## Discussion

Our qualitative investigation generated an account of HCPs’ experiences of the (patchy) reintroduction of PCSP, with a heavy reliance on telephone-based communications for PCSP conversations, following an initial cessation of PCSP activity when practices were instructed to conduct all non-urgent consultations remotely due to risk of COVID-19. Systems to support preparation for PCSP conversations were disrupted and when practices gave patients ‘wide window’ time slots within which to expect a telephone call, HCPs often found people hard to get hold of or ‘not in the right headspace’ for a conversation focused on their health. The audio-only nature of telephone communication made it harder for HCPs to gauge how a person was doing and precluded real-time exchanges of written and visual information to support their discussions. In these circumstances, HCPs reported struggling to maintain the balance between patients’ and their own agendas, and to work collaboratively to develop goal setting and action plans, as well as to establish or sustain the intended relational ethos of PCSP. This was particularly challenging for nursing staff, who reported a general lack of training in remote consulting compared to GP colleagues. These challenges were experienced most acutely in work with patients living with more complex health conditions and in more disadvantaged social situations. Time spent working largely via telephone and in the variously constrained circumstances that endured beyond COVID-19 restrictions seems to have led some HCPs to lower their expectations of PCSP. HCPs acknowledged that telephone-based PCSP conversations could work well for patients who were managing and could communicate well, and with whom they had an established relationship (or at least had a previous face-to-face encounter). However, HCPs were keen to prioritise face-to-face appointments for those who needed them most. HCPs were also concerned to ensure that preparatory information gathering and sharing stages were functioning well for all, and that PCSP designated appointments were not reduced from their richly person-centred solution-focused ambitions to the kind of narrowly biomedical transactional encounters they were intended to replace.

### Strengths and limitations of study

This study adds to the limited research on telephone consulting in UK primary care, and it specifically addresses PCSP and telephone consulting for people living with long-term (and often multiple) health conditions in a changing primary care landscape. From the population of practices in England and Scotland where PCSP was being conducted, we secured a sample that included HCPs from different professional backgrounds, practising in geographically dispersed locations, serving patient populations with different socioeconomic and ethnicity profiles, and who had been using PCSP for varied lengths of time.

The study benefited from an in-depth qualitative dataset generated during a time of significant pressure on UK healthcare services in the wake of the COVID-19 emergency. Our team members brought familiarity with PCSP and long-term conditions from different health professional, patient and academic backgrounds to the discussions that supported our development of interpretive themes.

The main limitations are that we did not directly observe any telephone (or in person) PCSP conversations, we did not interview patients, and we did not attempt a larger quantitative study to estimate the prevalence and distribution of reported experiences and views. Also, while there were many subtle indications of variability in the extent to which HCPs had internalised and were able to enact the key principles of PCSP (beyond working through process steps), we did not attempt to assess and categorise or grade interpretations or skills relating to PCSP more formally, so can say little about how these may have moderated responses to the shift to telephone consulting.

Our study design only allowed us to examine the implications of conducting PSCP conversations by telephone in a pandemic context in which a number of other changes to healthcare practice occurred at around the same time (most tending to undermine PCSP processes) and many people, including HCPs, were experiencing more rather than fewer challenges to their health and wellbeing. Nonetheless, as we outline here, our analysis includes important learning for the future delivery of PCSP by remote approaches.

### Congruence with other studies of remote consulting

Our participants’ concerns about the adverse impacts of telephone consulting on rapport building and the relational aspects of consulting, and that people living with complex clinical conditions and/or social disadvantage were worst affected by the limitations of telephone consulting, are consistent with other evidence about the broader shift to remote healthcare consulting in UK general practice [10, 12, 15, 20–23]. The concerns about relationality and equality are particularly troubling for PCSP given the approach’s commitments to person-centredness and ambitions for HCPs and patients to enter conversations as equal partners.

### Challenges of person-centred care

Several studies in different parts of the world have illuminated how challenging it can be for health professionals to shift from narrow biomedically-focussed consultations to respectfully responsive and enabling person-centred conversations aimed at supporting people to self-manage and live well on their own terms with their long-term conditions [24–27]. Strong leadership, compatible healthcare systems and processes, and time and support to develop the requisite attitude and skillsets (including with practice and reflection) all play a part in fostering and sustaining approaches like personalised care and support planning. In our study, HCPs’ accounts of their experiences with PCSP around the introduction of COVID-19 restrictions and beyond revealed losses relating to PCSP-supporting systems and process and circumstance changes that – especially when practice leaders were not firmly committed to PCSP – weakened scope to enact PCSP conversations as intended. A shift to telephone consulting also posed challenges. While communication by telephone should not in and of itself preclude a person-centred approach, it seems more suitable for some kinds and elements of consultation than for others. Significantly for the ambitions of PCSP conversations, with more limited clues as to how a patient is feeling, including in response to a conversational partner’s questions and comments, HCPs can find it more difficult to be confident about fine tuning their contributions so each patient can experience them as respectful and enabling.

We saw several hints in some HCP’s accounts that their attitudinal responses to patients and handling of some situations were not entirely congruent with the assumptions of PCSP. In the context of interviews about telephone consulting through pandemic challenges and with practices under various pressures, these could well have been circumstantially influenced. They do, however, serve as a reminder that person-centredness depends partly on the mindset of those providing services.

### Implications for future telephone-based PCSP consulting

In both England and Scotland, there are plans to continue the expansion of remote consulting in the NHS [16, 17]. All the HCPs in our study emphasised the importance of now offering patients a telephone (or video if requested) appointment for their PCSP conversation, but there was also strong support for continuing to provide (or reinstating) face to face PCSP conversations for people who preferred those, especially for people who were somehow struggling with health and/or social problems. Some of the limitations associated with telephone consulting could be less prevalent and mitigated in video consulting, although fewer than 1% of UK general practice consultations occur by video [12] and a shift to video would raise further questions about how a digital divide likely further exacerbates social inequalities in healthcare and health.

Our findings resonate with other studies which have highlighted the extent to which a shift to remote consulting can leave many HCPs missing face-to-face interactions with people and feeling an increased cognitive burden [12, 21]. Many of the HCPs we interviewed valued the relational emphases of PCSP and derived professional satisfaction from what it can achieve for patients. To the extent that these positive experiences are undermined by systemic pressures towards shorter and telephone based PCSP conversations, and the loss of support for PCSP more generally, there may be important implications for workforce wellbeing and sustainability. The British Medical Association recently reported GP practices are “experiencing significant and growing strain with declining GP numbers, rising demand, [and] struggles to recruit and retain staff” [28]. If support to conduct PSCP can improve HCP fulfilment as well as benefit patients, its longer term value may exceed short term costs.

Ongoing changes in the primary care landscape mean careful attention is needed to implications for the future of PCSP and fulfilment of its person-centred aspirations. Given the likely continuation at least to some extent of PCSP conversations by telephone, there is a need to investigate patients’ experiences of the different modalities and to study more systematically how and why experiences and outcomes vary across patient groups (it will be important to attend to disparities in digital access and implications for inequities in healthcare [15, 29, 30]).

Scope to enhance HCP skills and confidence for conducting PCSP by telephone and/or video link, and to modify supporting organisational systems to enhance patients’ preparation for and experience of PCSP conversations also needs further attention. (Our study suggests areas likely to need attention but has not looked as far as interventional development).

## Conclusions

There are significant challenges to fulfilling some key activities and relational aspects of PCSP via telephone conversations. The use of telephone appointments generally can offer some additional flexibility for health professionals as they work through a range of tasks, and PCSP conversations by telephone can be an appropriate option for some patients, who may prefer its convenience. However, this study provides grounds for scepticism about how far telephone appointments are compatible with some person-centred models of working such as PSCP. Even if the processes of planning and preparation that enable PCSP are protected, the organisation and enactment of telephone calls risks undermining the broader ethos and benefits of PCSP. This is especially true for people who are socially disadvantaged and living with more complex health conditions.

### Electronic supplementary material

Below is the link to the electronic supplementary material.

Supplementary Material 1

Supplementary Material 2

Supplementary Material 3

## Data Availability

The datasets generated and analysed during the current study are not publicly available due to the agreement made with participants to protect individual professional privacy. They may be available from the corresponding author on reasonable request subject to approval from the University of Aberdeen.

## Abbreviations

[P]CSP: [Personalised] Care and Support Planning
HCP: Healthcare professional
SMS: Short Message Service
GP: General Practitioner
YOC(P): Year of Care (Partnership)
NP: Nurse Practitioner
PN: Practice Nurse
