# Supplementary material for: Telephone consulting for ‘Personalised Care and Support Planning’ with people with long-term conditions: a qualitative study of healthcare professionals’ experiences during COVID-19 restrictions and beyond
Source: BMC Prim Care. 2024 May 31;25:193. doi: 10.1186/s12875-024-02443-z (PMC11143770; doi:10.1186/s12875-024-02443-z)
Supplement: Supplementary file 1 — Supplementary Material 1 [file 12875_2024_2443_MOESM1_ESM.docx]

*Additional File 1: Participant Information Leaflet*


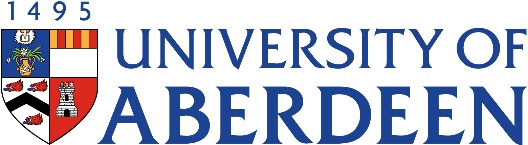

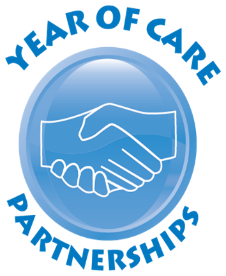
**
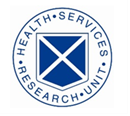
**

**Learning from healthcare professionals’ experiences of using remote consulting for ‘Care and Support Planning’ during COVID-19 (CASPER project).**

**Participant Information Sheet: Qualitative interview study**

**Chief Investigator: Professor Vikki Entwistle**

**IRAS ID: 300699**

You are being invited to take part in an interview for a research study. Before you decide it is important for you to understand why the research is being conducted and what it will involve. Please read this information carefully. Ask us if there is anything that is not clear or if you would like more information. Thank you for taking the time to read this and considering whether you wish to take part.

**What is the purpose of this study?**

# The main aim of this study is to investigate healthcare professionals’ experiences of the shift to using remote consulting for ‘Care and Support Planning’ (CSP) during COVID-19. It will consider how well the purposes of CSP can be achieved using remote technology (compared to face to face) and explore any adaptations that have been (or could be) made to CSP when using telephone and video consulting.

**Why have I been chosen?**

We are inviting healthcare professionals from general practices across the UK who had experience of using CSP in-person, as introduced by the Year of Care Partnership, prior to March 2020. We hope to interview approximately 16-25 healthcare professionals, including nurse practitioners, practice nurses, GPs and CSP trainers.

**What will I have to do if I take part?**

If you agree to take part, we will arrange a one-to-one interview (on-line) for between 45-60 mins, to talk about your experiences of using remote consulting for CSP. The interview will broadly focus on your experiences of conducting remote CSP consultations during COVID-19, compared to previous in-person interactions. In particular, we will ask you to reflect on what you think has worked well and what not so well with remote consulting, and to tell us about any adaptations that have been (or could be) made to CSP when using telephone or video consulting. To help us better understand, we will ask you to talk us through some examples to

illustrate your experiences. We will share an outline of the questions with you ahead of your interview.

The interviewer will be a trained researcher from the University of Aberdeen, and the conversation can take place at a convenient time for you via Microsoft Teams. The discussion will be video and audio-recorded, and transcribed by an accredited company external to the University of Aberdeen (with agreements in place for handling of personal data). All information which is collected about you during this study will be kept strictly confidential and data will be reported in a way which ensures anonymity.

**Do I have to take part in an interview?**

No. It is your decision about whether you wish to take part or not. If you do agree to take part and then change your mind, you can withdraw at any time without giving a reason. However, we will keep the information that we already have. To safeguard your rights, we will only use the minimum personally identifiable information possible.

**What are the risks and benefits of taking part in this interview study?**

We do not anticipate any risks to those taking part in an interview. We also do not anticipate there will be any direct benefit to you if you do take part, but you will be helping with research that YOCP will use to help develop training, guidance and support for health professionals using CSP, aiming ultimately to benefit patient care. As a small token of our appreciation, you will be reimbursed with an Amazon voucher (worth £25) for your time and contribution.

**What will happen to the results of the study?**

The preliminary findings from this study will be shared with contributing practice teams and broader professional contacts, including via Year of Care Programme online webinars. Feedback from these will be used to refine the analysis and inform the ongoing development of practical support offered by YOCP. They will also inform the development of a larger scale investigation of CSP consultations that includes attention to patients’ perspectives.

We also plan to report the findings in peer-reviewed journals, at scientific research meetings and we may use findings to support other research/teaching in the future. The information that we report (including quotes) will be anonymised (using pseudonyms) and would not identify you in any way.

**Will my taking part in this study be kept confidential?**

Your interview will be typed up by a University of Aberdeen approved transcribing company, who will treat your data confidentially. Audio files and transcripts will be stored securely on the University of Aberdeen server, with access restricted to the study team. All recordings and transcripts will be destroyed 6 years after the end of the study. Data sharing with the wider research team (outside of the University of Aberdeen) will be limited to anonymised interview transcripts and thematic analysis charts. Data will be shared securely with collaborators (colleagues at King’s College London and the NHS Year of Care Partnership) using the University of Aberdeen ZendTo secure encrypted service.

The University of Aberdeen is the sponsor for this study. All information which is collected about you during the research will be kept strictly confidential. We will only use the minimum personally identifiable information possible. The University of Aberdeen will act as the data controller for this study.

**How will we use information about you?**

We will need to use information from you for this research project. This information will include your initials, name, and contact details. People will use this information to do the research or to check your records to make sure that the research is being done properly.

People who do not need to know who you are, will not be able to see your name or contact details. Your data will have a code number instead. We will keep all information about you safe and secure. We will write our reports in a way that no-one can work out that you took part in the study. All recordings and transcripts will be destroyed 6 years after the end of the study.

**What are your choices about how your information is used?**

You can stop being part of the study at any time, without giving a reason, but we will keep information about you that we already have.

We need to manage your records in specific ways for the research to be reliable. This means that we won’t be able to let you see or change the data we hold about you.

If you agree to take part in this study, you will have the option to take part in future research using your data saved from this study.

**Where can you find out more about how your information is used?**

If you have any questions about the University’s handling of your information, you can contact the University Data Protection Officer in the following ways:

- by asking one of the research team
- by sending an email to [dpa@abdn.ac.uk](mailto:dpa@abdn.ac.uk)
- by ringing us on 01224  272596

Further information about the University’s Privacy Notice can be found here: <https://www.abdn.ac.uk/about/privacy>/research-participants-938.php

**What if something goes wrong?**

If you have a concern about any aspect of this study, you should ask to speak to the researcher who will do their best to answer your questions (sharon.mccann@abdn.ac.uk). If you remain unhappy and wish to complain formally, you can do this by contacting Research Governance at the University of Aberdeen ([researchgovernance@abdn.ac.uk](mailto:researchgovernance@abdn.ac.uk)).

**Who is organising and funding the research?**

The University of Aberdeen is sponsoring the research and the study is funded by the Wellcome Trust Institutional Strategic Support Fund (University of Aberdeen).

**What ethical and data permissions are in place?**

This study has been approved by The University of Aberdeen College Ethics Review Board. The organisation responsible for your personal information in terms of data protection legislation is the University of Aberdeen ([dpa@abdn.ac.uk](mailto:dpa@abdn.ac.uk)).

**What happens next?**

If you would like to take part, please contact the researcher by email [sharon.mccann@abdn.ac.uk] to arrange a convenient time to discuss the study further and set up the interview. Please note you will be asked to provide recorded verbal consent at the time of interview.

Thank you for reading this leaflet and for considering taking part in this study.

**Contact for further information:**

**Researcher: Dr Sharon McCann (University of Aberdeen)**

**Email: sharon.mccann@abdn.ac.uk**
