# Supplementary material for: Telephone consulting for ‘Personalised Care and Support Planning’ with people with long-term conditions: a qualitative study of healthcare professionals’ experiences during COVID-19 restrictions and beyond
Source: BMC Prim Care. 2024 May 31;25:193. doi: 10.1186/s12875-024-02443-z (PMC11143770; doi:10.1186/s12875-024-02443-z)
Supplement: Supplementary file 2 — Supplementary Material 2 [file 12875_2024_2443_MOESM2_ESM.docx]

*Additional File 2: Interview topic guide*

**1.To give me some background, can you describe how had you been using CSP before the pandemic?**

Probes:

-What were you trying to achieve/intention in your CSP interactions pre COVID? [clarify intent and purpose of CSP]

-Do you think the purpose or intention of CSP is different to other consultations? [clarify: If yes, in what ways and understanding of core features of CSP]

**2. How have you experienced the shift to conducting CSP using remote consulting?**

Probes**:**

- What were your initial thoughts about the feasibility of moving to remote consulting for CSP? [clarify: what shaped those views]

-How is conducting CSP by telephone (and/or video) different to how you did it before? Can you walk me through an example of a consultation that you think went differently than it might have done if conducted face to face? [clarify what has been different, why and for whom?

-How do you think the shift to remote consulting has directly and/or indirectly affected how you *prepare* and *conduct* CSP consultations? [clarify how, why]

**3. In your experience during remote CSP consulting, how does the purpose and structure of the CSP conversation work compared to previous in-person consultations?**

Probes:

-Do you feel what you are trying to achieve (or intentions behind your CSP interactions has changed in any way with remote consulting? [clarify: if yes, why, contexts, patients]

-In your experience, does the structure for CSP conversations work the same/worse/better compared to in-person consultations? [clarify what has changed, why and for whom]

Can you talk me through an example of where you think the CSP conversation has perhaps gone better than it might have done in-person? [clarify how, why, criteria, for whom]

Can you talk me through an example of where you think he CSP conversation has perhaps gone not so well as it might have done in-person? [clarify how, why, criteria, for whom]

**4. How easy or difficult has it been for you during remote CSP consulting to talk through and make a CSP plan with patients compared to how it was beforehand?**

Probes:

-Preferences around use of telephone/video technology- healthcare professional and/or patient driven? [clarify: why, for whom]

-Can you describe ways that you think the use of remote consulting has impacted on things such as building rapport, communication style, sharing ideas and give me any examples?

-Can you talk me through an example illustrating some of the complexities of trying to make a CSP plan over the telephone/video? [clarify: why, for whom]

**5. What adaptations have you made (or do you think could perhaps be made) to CSP when using remote consulting?**

Probes:

-What would you say have been the biggest benefits in using remote consulting for CSP compared to in-person consultations? Can you give me an example of that? [clarify: why, for whom]

-To help sum up, in your experience what aspects of the CSP conversation using remote technology can ‘feel lost’ or not work as well compared to in-person CSP conversations? Can you give me a few examples of this? [clarify for why, for whom]

**6. How do you tend to ‘judge’ whether you feel have done a ‘good job’ with a CSP or count it as a ‘successful’ CSP consultation?**

Probe:

-Has this changed with the shift to remote consulting or in the context of the pandemic? [clarify: if yes, how, why and for some patients but not others]

**7. Since using remote consulting for CSP, would you want to continue with this approach for CSP beyond the pandemic?**

Probes: -

-What kind of things would you want to change about CSP to enable you to continue with remote consulting for CSP?

-Has your personal commitment to what’s involved in CSP changed in any way during the pandemic? [clarify: yes/no, why]

**8. How do you think the shift to remote consulting has impacted on patients’ experiences of CSP?**

Probes:

- In what ways do you think the move to remote CSP consulting has impacted on patients regarding communication styles, relational aspects, rapport building, shared decision-making collaborative goal setting, planning & reviewing? [clarify: how, why, for whom]

-How well do you think your patients have adapted to this change of using remote consultations for CSP? [clarify: do they ask patients and what they say?]

-When you are thinking about what counts as ‘success’ or a ‘good job’ in your CSP interactions with patients, how does that ‘map’ onto how your patients might think themselves and their preferences?

**9. Is there anything you were hoping to talk about today that we have not covered?**

**10.** Can I check if it would be OK for the information collected during our interview today (i.e. anonymised views) to be used to support other research/teaching in the future and shared anonymously with other researchers/students? We have not added information here regarding the use of anonymised quotes in publications/presentations as it is already outlined in the consent process at the beginning of this interview.
