# Supplementary material for: Telephone consulting for ‘Personalised Care and Support Planning’ with people with long-term conditions: a qualitative study of healthcare professionals’ experiences during COVID-19 restrictions and beyond
Source: BMC Prim Care. 2024 May 31;25:193. doi: 10.1186/s12875-024-02443-z (PMC11143770; doi:10.1186/s12875-024-02443-z)
Supplement: Supplementary file 3 — Supplementary Material 3 [file 12875_2024_2443_MOESM3_ESM.docx]

*Additional File 3: CASPER summary chart example*

| **PC 003**  NP & CSP trainer (5-6 yrs) | “.. it was a more **holistic** picture, not just around the results; it was **much more about how are you, how well are you feeling, what’s happening, what are you hoping for or what are the things that are worrying you**... And, then ideally, and I use all these terms with some caution, **make a plan about what would help and going forward,** what would be a good thing to do.” p1  [highlighting how ‘timing’ around sequencing made this process less than ‘ideal’ in reality, see under sec b]  Compares previously **pts not getting results in advance of appt** was a bit like ‘**going to the headmaster for them to read your report to you” p4**  “But the thing about the CSP was, that the **whole implementation of somebody getting some information, getting some results**, getting something to prompt thinking, beforehand, so that they felt they at least they **had some leverage**, seemed to me the thing that we’d been, we’d been looking for it.” P4  CSP as **‘experts coming together…**fitted very much with our concept of **people being enabled.**.”P5 [broad understanding of CSP and its underlying principles for HCPs & Pts]  “I’m not sure that we’ve been doing CSP long enough, necessarily, for some people to actually see that, **they can, change their health, they can address some things, they can feel that they are empowered**..” pg 19 | Includes range of LTC’s , invite month of birth for review, info gathering followed by CSP conversation appt 2 weeks later.  Pre Covid issue - **“Sequencing”** problem  - **Pt not always getting results in time** for their 2^nd^ appt ; and/or  -Pt phones up practice for a **medication review** as have run low on medication, needs new prescription, sees nurse to discuss **‘out with CSP loop’** or asked to spk to GP about results and so sometimes **‘losing their place in scheme of things**.” P2,3  Initial **concerns about shift** to telephone consulting “..worried that we would lose, all the quality..”p9  Issues have improved now as they are **running so behind** with everything, so pt will have time to get results and read through before speaking to HCP.  **Telephone consults, very little video offered.** – during shift to remote found that approx. 50% of their patients didn’t have email/**smartphone access** or had to rely on family member to help them access it, struggling with texts etc. P8  **Pt confusion –** sometimes thinking ‘blood testing was *the* year of care and didn’t quite understand why they had a second appts and so **didn’t turn up for that one,[**2^nd^ part] and that happened quite a bit.” P2  **Questions use of ‘bits of paper’** and ‘writing letters’ tp pts as people don’t always read them or **’don’t engage** much with written material’ p3  **Lack of GP involvement** - there is such a shortage of doctors…the doctors really aren’t engaged in it at all, and I think that’s a mistake, we have.pg ” Pts referred to GP if needing meds review for depression or specialist input for complex cases.  **Role of administrator in CSP delivery –** having a senior adm go on the training engaged them and **‘made a huge difference’** in organising admi side – ‘**she understood the point of it’p4** | Highlights importance of **losing cues** around seeing how pt walks, general demeanour, mood, facial expressions, body language etc which cannot be picked up on telephone. -Has to be much more **‘alert’** to words/phrases/ tone of voice to try and pick up on things  **Visuals**  Misses not being able to **show patients on scree**n or print off things for them so they can see results/trends etc. Talks about this being an important **‘dimension’** in the context of the CSP conversation.p8. Easier to deal with **F2F printing off** p12  Highlights potential prob if pt on telephone with results letter- ‘**difficult for the consultation to be quite so holistic because, they’re on their own with that sheet…they tend to be focused on it.” P12**  Currently, feels as though CSP is ‘**drifting’** to a ‘default’ of being a **‘medication review’**  **Use of platforms to enable HCP’s gather pt data** via questionnaires v positive – eg able to send asthma questionnaires, depending on high/low score pts would be recalled p6 and/or at least give HCP some information ahead of appt p9  **CSP structure** seems to work for telephone, can be harder with lack of visuals  **Playing detective** - “you get **clues** from people when you see them, that actually they don’t give a sausage what their HBA1C is at the moment, because there’s something much more important that’s bothering them. But that’s a little bit less easy to pick up, on the phone, or they just **let a phrase drop** when they’re just about to finish the call, and it’s very difficult to pick that up again. Whereas if they’re at the door, you can say, “Gosh, that sounds difficult. You know, do you want to just have a little explore about that, because that sounds really problematic?”  …you have to be **alert..”**pg13  In conversation using more techniques to ‘**draw out more information’** p17 eg open ended/can you tell me more about that/some people have said x to me, can you? Pts will ‘**drop cues’** if listening well p17  **Adjustments made have led to more of a focus on ‘clinical points’** as getting less of a picture remotely “I find it more difficult to take a steer from them, I think that would be the truth really.” P16 | **More engagement via remote** consulting has helped engage certain pt groups eg younger adults which has historically been difficult  Feels telephone consulting is only providing a ‘**partial picture’** and worries about what she is **‘missing’** when not seeing patients in clinic.p18  **Loss of visuals** and reflection on how sometimes CSP **conversation can be limited** if pt on telephone with results letter, hard to think more broadly p13  Perceives remote consulting as **‘less holistic’ only gives ‘partial picture’ – p8, 13**  F2F feeling better for pt ‘..from their point of view, I think it’s **easier to show, that you’re really listening when you’re face to face** ..” p8  Concerned there has been **no forward planning** about CSP future post covid p13  Feels plans with pts have become **more ‘clinical’** as less able to ‘get the full picture’ over the telephone.  Some Pt’s seeing CSP remote consulting as a ‘**medication review’** and **‘less about them.****’** “..if we don’t take a hold and try and improve, try and focus on making sure that we maintain the centrality of CSP, I think there is **a risk, that it will become a brisk medication r**eview on the telephone, and focus more and more on the clinical aspects, on **the strictly measurable clinical aspects** I mean.” P16  [awareness of potential impact on some pts no longer feeling CSP focussed on them and more about how it use to be prev = medication review.]  Worries that telephone consulting has left some **patients feeling ‘less committed’** about their health condition and perception that the **HCP is possibly less committed as ‘just giving them a ring’ instead of F2F**. – also fuelled by **media reporting** on lack of access to GP’s p16  Currently, feels as though CSP is ‘**drifting’** to a ‘default’ of being a **‘medication review’ p13/14**  Telephone consulting can feel more ‘**convenient’ for HCP’s** driving their prefs p15 [provider driven care v pt driven care]  Tele consulting for those unable/unwilling to venture in GP’s it at least provide access for them.  **Future** – accepts should be more ‘options’ offered to patients about how they would like their CSP to take place i.e video/telephone/face to face.  Describes feeling more positive about telephone consulting than she thought she would, but that all patients who wish to be seen should be seen face to face as the default. [shifting narratives from initial lockdown..] “.. I’m less negative than I was, before we had to do it. … I would be glad to see everybody who thought it would be helpful to come, so I’d rather that be the default mode, but I don’t think we should discard those **options.** “ p19  Gives consideration to CSP via **group consultations** as similar format has worked well in practice for diabetes groups p10-11, but acknowledges **potential ‘gains & losses’** of such an approach p11  **Judgement criteria for a good/less good CSP** -p18 based on the **pt initiating some goal/chang**e - “something comes from them.” Feels less of a good CSP when conversation is ‘**very monosyllabic**” p18  Importance of getting **more GP involvement** – “I think I would have liked some of them to have been on the training because they’d understand what we were driving at more..”.”p1 |
| --- | --- | --- | --- | --- |
